# Supplementary material for: The Effects of Exercise Habit on Albuminuria and Metabolic Indices in Patients with Type 2 Diabetes Mellitus: A Cross-Sectional Study
Source: Medicina (Kaunas). 2022 Apr 23;58(5):577. doi: 10.3390/medicina58050577 (PMC9143753; doi:10.3390/medicina58050577)
Supplement: Supplementary file 1 [file medicina-58-00577-s001.zip › medicina-1622290-supplementary.pdf]

Supplementary Table S1.

GLM regression coefficients- albuminuria (mg/L)

Model	Unstandardized Coefficients B	Unstandardized Coefficients Std. Error	Standardized Coefficients Beta	t	*p*	95% CI for B Lower Bound	95% CI for B Upper Bound
1 (Constant)	-120.4	88.87		-1.36	0.177	-295.9	55.07
HbA1c (%)	21.44	12.58	0.133	1.70	0.090*	-3.42	46.29
1 (Constant)							
FPG (mg/dl)	0.71	0.31	0.177	2.27	<.05**	0.092	1.330
1 (Constant)	-94.72	16.48		-5.75	<.05**	-127.3	-62.18
TG (mg/dl)	0.94	0.10	0.588	9.18	<.05**	0.74	1.15
1 (Constant)	132.03	50.51		2.61	<0.05**	32.27	231.8
HDL (mg/dl)	-2.17	1.04	-0.16	-2.08	<0.05**	-4.22	-0.11
1 (Constant)	-181.1	64.9		-2.79	<0.05**	-309.3	-52.77
FPG (mg/dl)	0.39	0.27	0.096	1.46	0.146	-0.14	0.91
TG (mg/dl)	0.95	0.11	0.592	8.68	<.05**	0.73	1.16
HDL (mg/dl)	0.74	0.92	0.055	0.80	0.424	-1.08	2.56
1 (Constant)	-173.0	91.0		-1.90	0.059	-352.8	6.74
HbA1c (%)	7.21	10.5	0.045	0.69	0.494	-13.56	27.99
TG (mg/dl)	0.95	0.11	0.595	8.66	<.05**	0.74	1.17
HDL (mg/dl)	0.56	0.92	0.042	0.61	0.543	-1.25	2.36
1 (Constant)	-179.7	91.0		-1.98	0.050	-359.3	0.028
HbA1c (%)	-0.27	12.0	-0.002	-0.02	0.982	-23.97	23.45
FPG (mg/dl)	0.39	0.30	0.097	1.28	0.201	-0.21	0.99
TG (mg/dl)	0.95	0.11	0.592	8.62	<.05**	0.73	1.17
HDL (mg/dl)	0.74	0.92	0.055	0.80	0.425	-1.09	2.56

* Difference between the exercise and non-exercise groups ($p < 0.1$). ** Difference between the exercise and non-exercise groups ($p < 0.05$).

Abbreviations: FPG: fasting plasma glucose; GLM: general linear model; HDL: high-density lipoprotein; HbA1c: glycated haemoglobin; TG: triglycerides.
